# Development of a nomogram for predicting the risk of left ventricular diastolic function in subjects with type-2 diabetes mellitus

**DOI:** 10.1007/s10554-021-02338-5

**Published:** 2021-11-16

**Authors:** Yuan Chen, Meng Yu, Yalin Lan, Fei Feng, Chengyan Jiang

**Affiliations:** Department of Endocrinology, The First People’s Hospital of Zunyi, The Third Affiliated Hospital of Zunyi Medical University, Zunyi, Guizhou Province China

**Keywords:** Epicardial fat, Left ventricular, Diastolic function, Type-2 diabetes mellitus

## Abstract

Left ventricular diastolic dysfunction (LVDD) can be affected by many factors, including epicardial adipose tissue (EAT), obesity and type-2 diabetes mellitus (T2DM). The aim of this study was to establish and validate an easy-to-use nomogram that predicts the severity of LVDD in patients with T2DM. This is a retrospective study of 84 consecutive subjects with T2DM admitted to the Endocrinology Department, the First People’s Hospital of Zunyi City between January 2015 and October 2020. Several echocardiographic characteristics were used to diagnose diastolic dysfunction according to the 2016 diastolic dysfunction ASE guidelines. Anthropometric, demographic, and biochemical parameters were collected. Through a least absolute shrinkage and selection operator (LASSO) regression model, we reduced the dimensionality of the data and determined factors for the nomogram. The mean follow-up was 25.97 months. Cases were divided into two groups, those with LVDD (31) and those without (53). LASSO regression identified total cholesterol (Tol.chol), low-density lipoprotein (LDL), right ventricular anterior wall (RVAW) and epicardial adipose tissue (EAT) were identified as predictive factors in the nomogram. The ROC curve analysis demonstrated that the AUC value for most clinical paramerters was higher than 0.6. The nomogram can be used to promote the individualized prediction of LVDD risk in T2DM patients, and help to prioritize patients diagnosed with echocardiography.

**Supplementary Information:**

The online version contains supplementary material available at 10.1007/s10554-021-02338-5.

## Background

Under normal conditions, epicardial adipose tissue (EAT) can release massive adrenomedullin as a potential peptide, a potent vasodilator. Angiotensin II induced oxidative stress is antagonized by adrenomedullin which inhibits release of endothelial cell apoptosis and endothelin-1, and suppresses migration and proliferation of vascular smooth muscle cells (VSMCs) [1]. Stefan Möhlenkamp reported that among people in general, EAT is related to fatal and nonfatal coronary events, but not affected by traditional cardiovascular risk factors [2]. In addition, EAT is also believed to affect left ventricular (LV) function [3–5]. Minkyung Kim [5] found greater EAT in patients with metabolic syndrome (MetS) and it has significant correlation with LV dysfunction in those with MetS. However, no such related studies have been published about the relation of EAT and LV function in subjects with type-2 diabetes mellitus (T2DM).

Adipocytes from epicardial fat are considered to be great association of echocardiographic epicardial fat thickness, a common situation in obese patients with T2DM [6]. The analogic relation between intra-abdominal visceral fat and EAT was shown in echocardiographic studies [7, 8]. As compared to normoglycemic subjects, Yang et al. [9] demonstrated that pericardial adipose tissue (PAT) volume was much higher in diabetics. The presence of T2DM might be another influencing factor of LV diastolic dysfunction.

The correlation of EAT with either left ventricular diastolic dysfunction (LVDD) or T2DM have been separately reported. Yet, the association of EAT and LV diastolic dysfunction in the occurence of T2DM has not been studied in a large Chinese population. Thus, we intended to evaluate the relevance between clinical parameters and LVDD in a population of cases with T2DM using two-dimensional echocardiography. Nevertheless, no previous studies have reported a nomogram used in subjects with T2DM.

The study was aimed to develop and validate an easy-to-use nomogram depended on clinical features of demographic, anthropometric and biochemical parameters to predict the risk of LVDD in patients with T2DM. It was expected that the nomogram could be applied in the clinic to increase the LVDD diagnoses in subjects with T2DM by identifying patients at high risk of LVDD who should be referred for treatment.

## Methods

### Study population

Subjects were included in the retrospective longitudinal control cohort study evaluating abnormally elevated blood glucose, accompanied by lack of insulin for its pathogenicity in terms of cardiac function heart failure.

The cohort consisted of 38 men and 46 women aged ≥ 18 years and ≤ 75 years, who were newly diagnosed with T2DM between January 2015 and October 2020 at the First People’s Hospital of Zunyi City in China. Patients were eligible for inclusion if they gave autonomous informed consent. Their medical and imaging records were reviewed retrospectively. The T2DM was diagnosed according to the American Diabetes Association (ADA) criteria [10].

Exclusion criteria were pregnancy, cancer, using thyroid or steroids hormones, congenital heart disease, diabetes mellitus type 1, diagnosed rheumatoid, inflammatory bowel, genetic diseases, mental disorders, or addiction to drugs or alcohol. The research was approved by the ethics committee and investigation complies with the principles stated in the Declaration of Helsinki [11].

### Collection of clinical data

The age and gender of participants were recorded. BMI was calculated as weight in kilograms dividing by the square of height in meters. Blood pressure was measured three times, with a mercury sphygmomanometer after subjects had been rest quietly for 10 min. After an overnight fast, serum appearance samples were obtained and immediately stored for delivering in the second morning. We used standard laboratory procedures to estimate and calculate the following biochemical parameters: glycated hemoglobin (HbA1C), high-density lipoprotein (HDL), low-density lipoprotein (LDL), total cholesterol (T.chol), fasting glucose, fasting insulin.

### Echocardiography

All participants underwent a standard transthroacic ultrasound equipment (TTE) (Vivid 7, GE Medical Systems, Milwaukee, WI) lying on left lateral side. According to American Society of Echocardiography guidelines [12], the cardiac parameters were measured: LV end-diastolic, end-systolic dimensions, LV end-diastolic wall thickness, apical four-chamber area and parasternal long axis diameter. The identification of epicardial fat is the echo-free space between the visceral layer of the pericardium and the outer wall of the myocardium. It is measured on the free wall of the right ventricle at end-diastole using a two-dimensional parasternal long-axis view [7].

### Left ventricular diastolic dysfunction

LVDD as defined by the following criteria: (I) septal e′ < 7 cm/ sec, (II) lateral e′ < 10 cm/sec, (III) peak TR velocity > 2.8 m/sec. (IV) average E/e′ ratio > 14 [13] (V) LA volume index > 34 mL/m^2^. LVDD is identified if more than two parameters meet the above cutoff values, according to standard the ASE and EACVI (European Association of Cardiovascular Imaging) updated LVDD guidelines [14].

### Epicardial adipose tissue

EAT thickness was described as the echofree space consist of the fat depot on the external surface of myocardium and outside the visceral layer of the pericardium [15, 16]. It was measured on the free wall of the right ventricle during 3 cardic end-systole from the parasternal long-axis view. So as to ensure the accuracy and scientificity, the analyse uesd average value of three cardiac cycles.

### Statistical analysis

Continuous variables are expressed as frequency or the mean ± standard deviation SD. Chisquare analysis and unpaired Student’s t-test were chosen to compare variables (categorical and continuous variables). The primary outcome of this study was to evaluate the influence of T2DM in EAT changes. The glmnet package in R was used to perform selection operator (LASSO) logistic regression and least absolute shrinkage. We construct a nomogram through significant factors identified by LASSO model, helping identify patients at risk of LVDD [17]. According to Copas’s proposal [18], model calibration was assessed to produce calibration plots that graphically elaborate the links between the observed and predicted probabilities of LVDD. All statistical analyses were performed using R 4.0.2 (TUNA Team, Tsinghua University, China) and statistical software package IBM SPSS (version 23.0). Values of P < 0.05 were considered statistically significant.

## Results

### Baseline characteristics

Baseline demographic and clinical characteristics of subjects are shown in Table 1. Among both groups, no statistical differences were found in gender, age, diastolic blood pressure (DBP), Weight, body mass index (BMI), triglyceride, LDL cholesterol, glycated hemoglobin (HbA1C). The mean age of all patients was 58.58 ± 2.24 years old, and 14 subjects were male in the patients with LVDD. The mean diastolic blood pressures were 78.06 ± 5.76 mmHg, respectively. The mean BMI was 25.27 ± 1.06 kg/m^2^ in the overall population. According to the above‐defined criteria, 31 subjects were defined as group with LVDD and 53 subjects are with normal LVDF.

**Table 1:** Baseline characteristics

Parameter	Total	LVDD	Normal	p-value
	n = 84	n = 31	n = 53	
Male, n (%)	38 (45.24)	14 (45.16)	24 (45.28)	1
Age, yrs	58.76 ± 1.97	58.58 ± 2.24	58.86 ± 1.79	0.44
SBP, mmHg	131.48 ± 10.59	136.87 ± 8.56	128.32 ± 10.38	0.00032
DBP, mmHg	78.06 ± 5.76	77.58 ± 5.70	78.34 ± 5.77	0.46
BMI, kg/m^2^	25.27 ± 1.06	25.16 ± 1.17	25.33 ± 0.98	0.57
Triglyceride, mg/dL	2.29 ± 0.38	2.97 ± 0.87	1.86 ± 0.25	0.17
T. cholesterol, mg/dL	4.42 ± 0.42	4.89 ± 0.22	4.15 ± 0.23	2.8e − 14
LDL cholesterol, mg/dL	2.61 ± 0.14	2.71 ± 0.13	2.55 ± 0.11	2.5e − 06
HDL cholesterol, mg/dL	1.07 ± 0.12	0.98 ± 0.05	1.13 ± 0.11	4.1e − 09
HbA1C, %	8.77 ± 0.66	8.25 ± 0.58	9.07 ± 0.50	6.4e − 08
Fasting glucose, mg/dL	8.90 ± 0.81	9.20 ± 1.11	8.72 ± 0.49	0.041
Fasting insulin	18.68 ± 3.94	19.47 ± 4.19	18.21 ± 3.71	0.23
Echocardiography				
Systolic function (EF, %)	61.06 ± 6.42	62.19 ± 5.44	60.40 ± 6.84	0.2
EAT, mm	3.59 ± 0.47	4.08 ± 0.33	3.30 ± 0.23	5.8e − 13
RVAW, mm	4.08 ± 0.34	4.35 ± 0.31	3.92 ± 0.24	2e − 07
RVLW, mm	4.26 ± 0.69	3.92 ± 0.27	4.47 ± 0.78	0.0017
LVPW, mm	2.94 ± 0.23	2.98 ± 0.27	2.92 ± 0.20	0.26

*BMI* body mass index, *DBP* diastolic blood pressure, *EAT* epicardial adipose tissue, *HbA1C* glycated hemoglobin, *HDL* high-density lipoprotein, *LDL* low-density lipoprotein, *SBP* systolic blood pressure, *T.chol* total cholesterol. *RVAW* right ventricular anterior wall, *RVLW* right ventricular lateral wall, *LVPW* left ventricular posterior wall

### Clinical and ultrasonic inspection parameters in groups with LVDD and normal LVDF

**Fig. 1:**
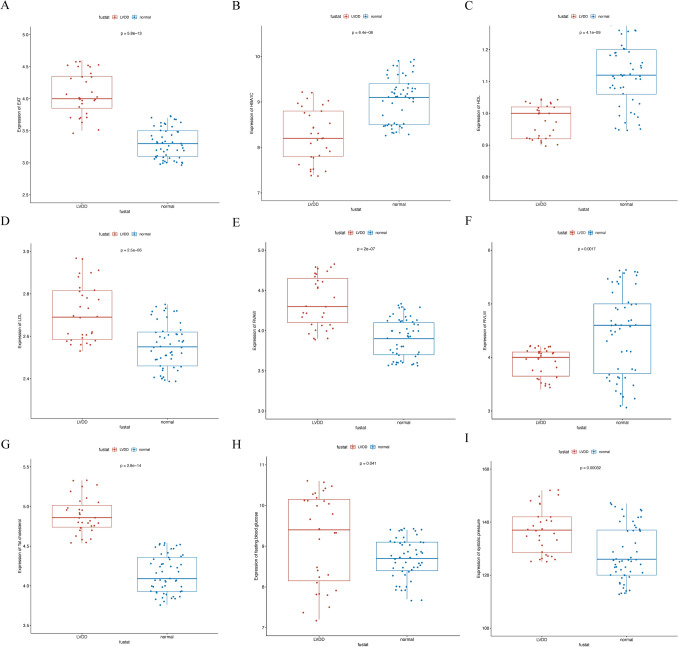
Patients with different clinicopathological features (including EAT, HbA1C, HDL, LDL, RVAW, RVLW, T. chol, fasting blood glucose and systolic pressure) had different levels of diastolic function. *EAT* epicardial adipose tissue, *HbA1C* glycated hemoglobin, *HDL* high-density lipoprotein, *LDL* low-density lipoprotein, *RVAW* right ventricular anterior wall, *RVLW* right ventricular lateral wall, *T.chol* total cholesterol, *SBP* systolic blood pressure

The comparative analysis of the clinical parameters is shown in Table 1. Among subjects with LVDD, systolic blood pressure (SBP) was 136.87 ± 8.56 mmHg, compared to 128.32 ± 10.38 mmHg in the patients with normal LVDF (p = 0.00032). Figure 1 the T. cholesterol, LDL cholesterol, fasting glucose, RVAW and EAT were significantly higher in subjects with LVDD (4.89 ± 0.22 vs 4.15 ± 0.23 mg/dL, 2.71 ± 0.13 vs 2.55 ± 0.11 mg/dL, 9.20 ± 1.11 vs 8.72 ± 0.49 mg/dL, 4.35 ± 0.31 vs 3.92 ± 0.24 and 4.08 ± 0.33 vs 3.30 ± 0.23 mm in subjects with LVDD and normal LVDF, p < 0.05). (Fig. 1, Table 1) There is no statistical significance in DBP, Triglyceride, fasting insulin, systolic function (EF, %), and left ventricular posterior wall (LVPW) between two groups.

### Correlation of clinical and ultrasonic inspection parameters in two groups

**Fig. 2:**
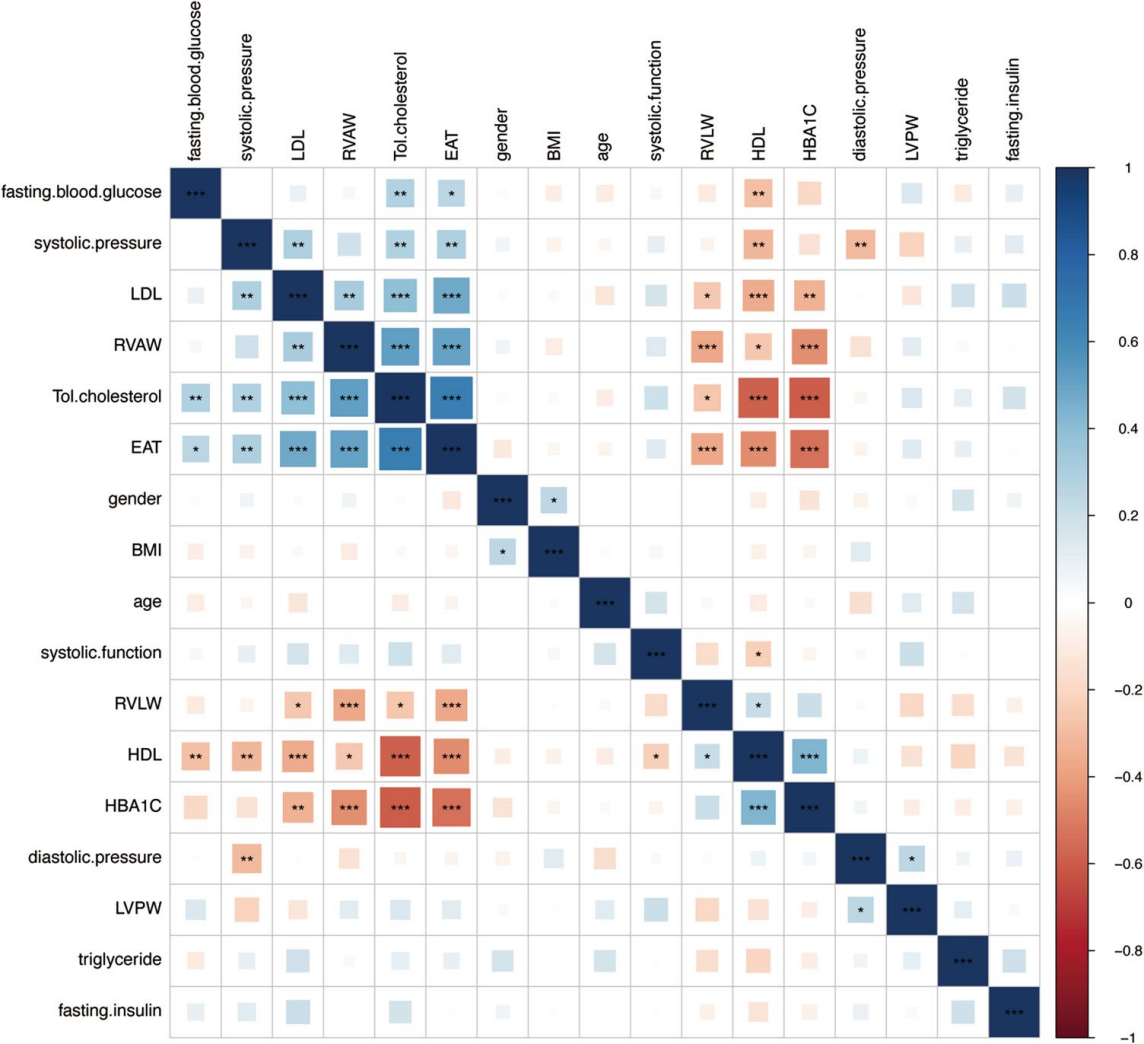
Heatmap of the correlations between 12 clinical characters and 4 ultrasonic inspection parameters. *LDL* low-density lipoprotein, *RVAW* right ventricular anterior wall, *EAT* epicardial adipose tissue, *BMI* body mass index, *RVLW* right ventricular lateral wall, *HDL* high-density lipoprotein, *HbA1C* glycated hemoglobin, *LVPW* left ventricular posterior wall

Using bivariate analysis to clarify the association between various parameters and LV diastolic function (Table 2). In subjects with LVDD, EAT was significantly related to HDL (r = 0.44, p = 0.01). In subjects with normal LVDF, fasting insulin (r = − 0.28, p = 0.04) showed significant correlation with EAT. Additionally, the correlations between 12 clinical characters and 4 ultrasonic inspection parameters were also displayed in Fig. 2 (Table 3).

**Table 2:** Bivariate correlation analysis with EAT

Parameter	LVDD		Normal	
	Correlation coefficient	p-value	Correlation coefficient	p-value
gender	− 0.34	0.06	− 0.06	0.65
age	− 0.13	0.48	0.13	0.34
systolic function (EF, %)	− 0.28	0.13	0.22	0.11
SBP	0.08	0.68	− 0.13	0.36
DBP	0.02	0.92	− 0.02	0.87
BMI	0.02	0.94	0.03	0.82
triglyceride	− 0.14	0.46	0.07	0.64
T. chol	− 0.31	0.09	0.12	0.39
LDL	0.06	0.75	0.06	0.68
HDL	0.44	0.01	0.06	0.66
HbA1C (%)	0.05	0.79	− 0.26	0.07
fasting blood-glucose	0.10	0.58	− 0.07	0.59
fasting insulin	− 0.08	0.64	− 0.28	0.04
RVAW	− 0.09	0.61	0.14	0.33
RVLW	0.09	0.64	− 0.20	0.16
LVPW	0.06	0.75	− 0.03	0.84

*BMI* body mass index, *DBP* diastolic blood pressure, *EAT* epicardial adipose tissue, *HbA1C* glycated hemoglobin, *HDL* high-density lipoprotein, *LDL* low-density lipoprotein, *SBP* systolic blood pressure, *T.chol* total cholesterol. *RVAW* right ventricular anterior wall, *RVLW* right ventricular lateral wall, *LVPW* left ventricular posterior wall

**Table 3:** LASSO feature regression model showing coefficient associated with LVDF

Parameters	Coef
T. chol	0.90
LDL	0.50
RVAW	0.25
EAT	0.91

*T. chol* total cholesterol, *LDL* low-density lipoprotein, *HbA1C* glycated hemoglobin, *RVAW* right ventricular anterior wall, *EAT* epicardial adipose tissue

### Factor selection for the predictive model

**Fig. 3:**
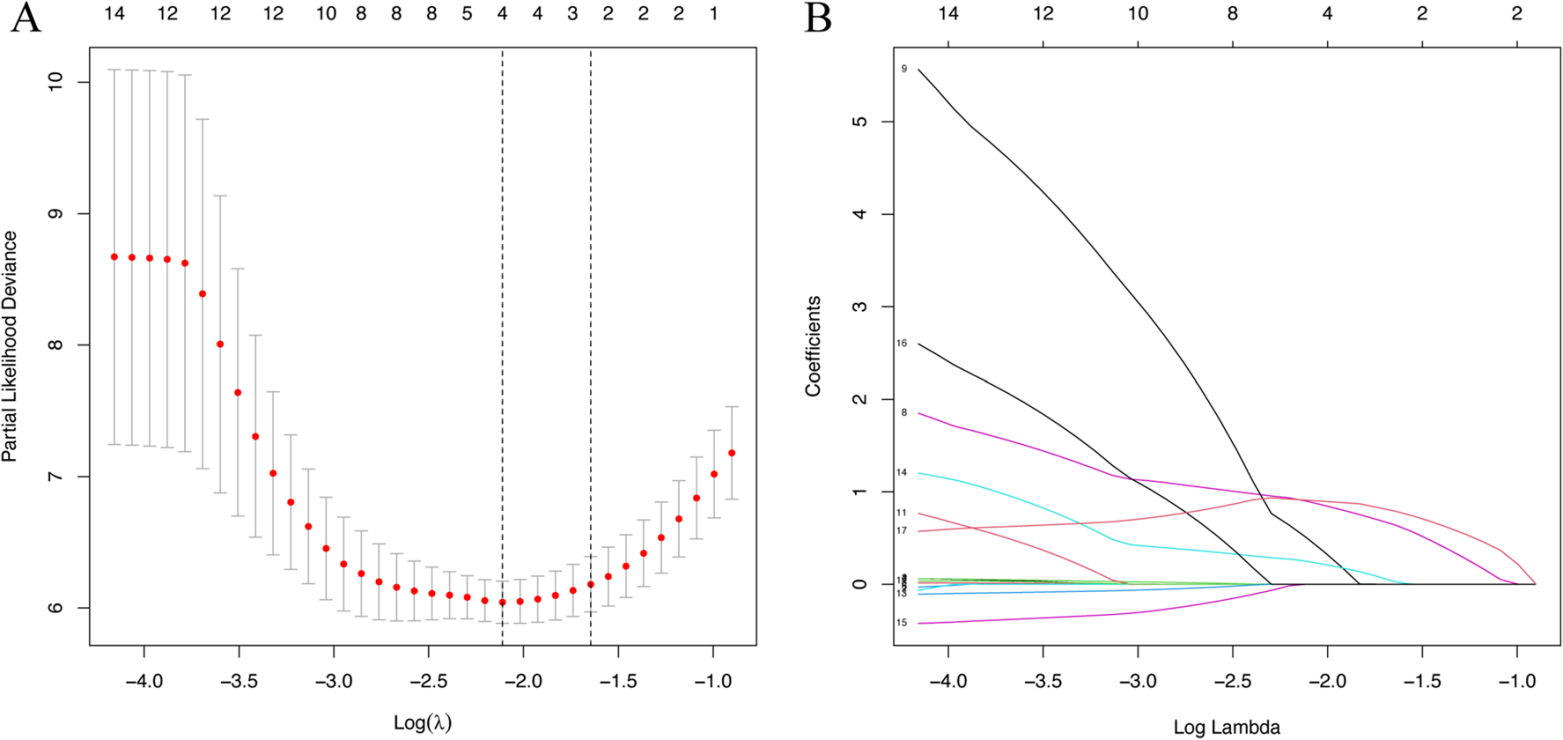
LASSO feature selection model. **A** LASSO coefficients of 14 candidate variables. **B** Identification of the optimal penalization coefficient (λ) in the LASSO model was achieved by tenfold cross-validation and the minimum criterion. The left vertical line represents the minimum error, and the right vertical line represents the cross-validated error within 1 standard error of the minimum. *LASSO* least absolute shrinkage and selection operator

**Fig. 4:**
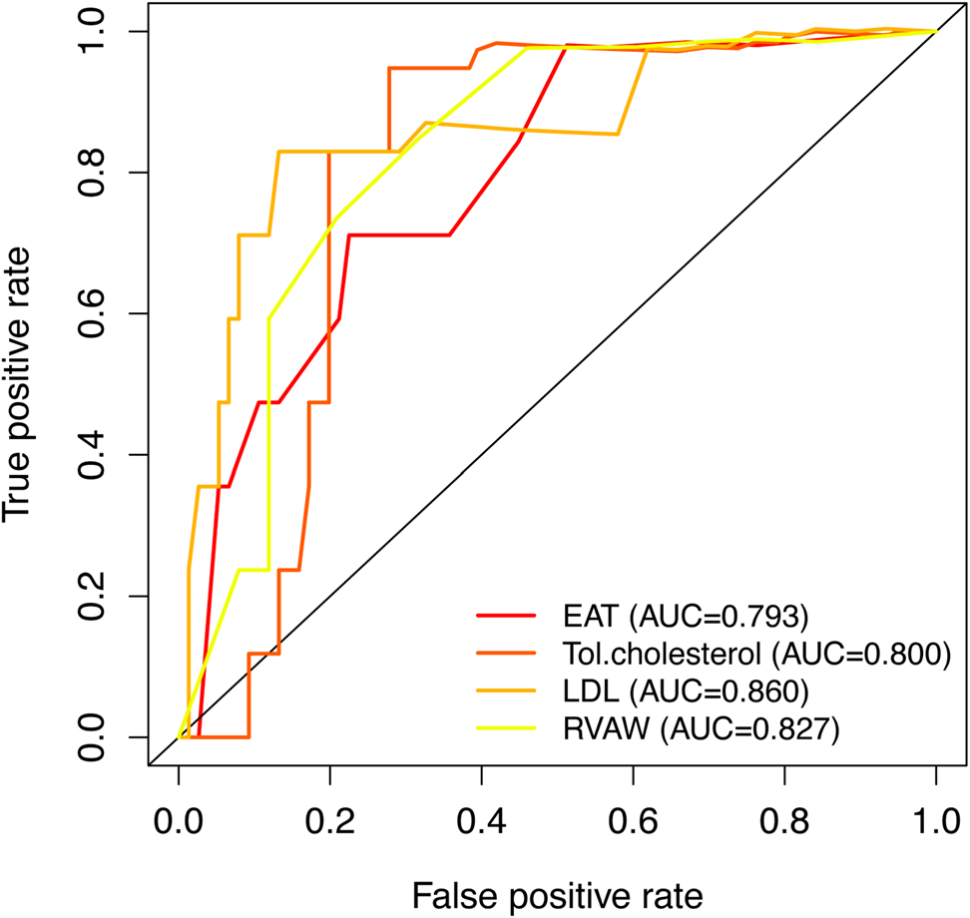
Time-dependent receiver operating characteristic (ROC) curves for the nomogram. EAT, Tol.chol, LDL and RVAW. *EAT* epicardial adipose tissue, *T.chol* total cholesterol, *LDL* low-density lipoprotein, *RVAW* right ventricular anterior wall

**Fig. 5:**
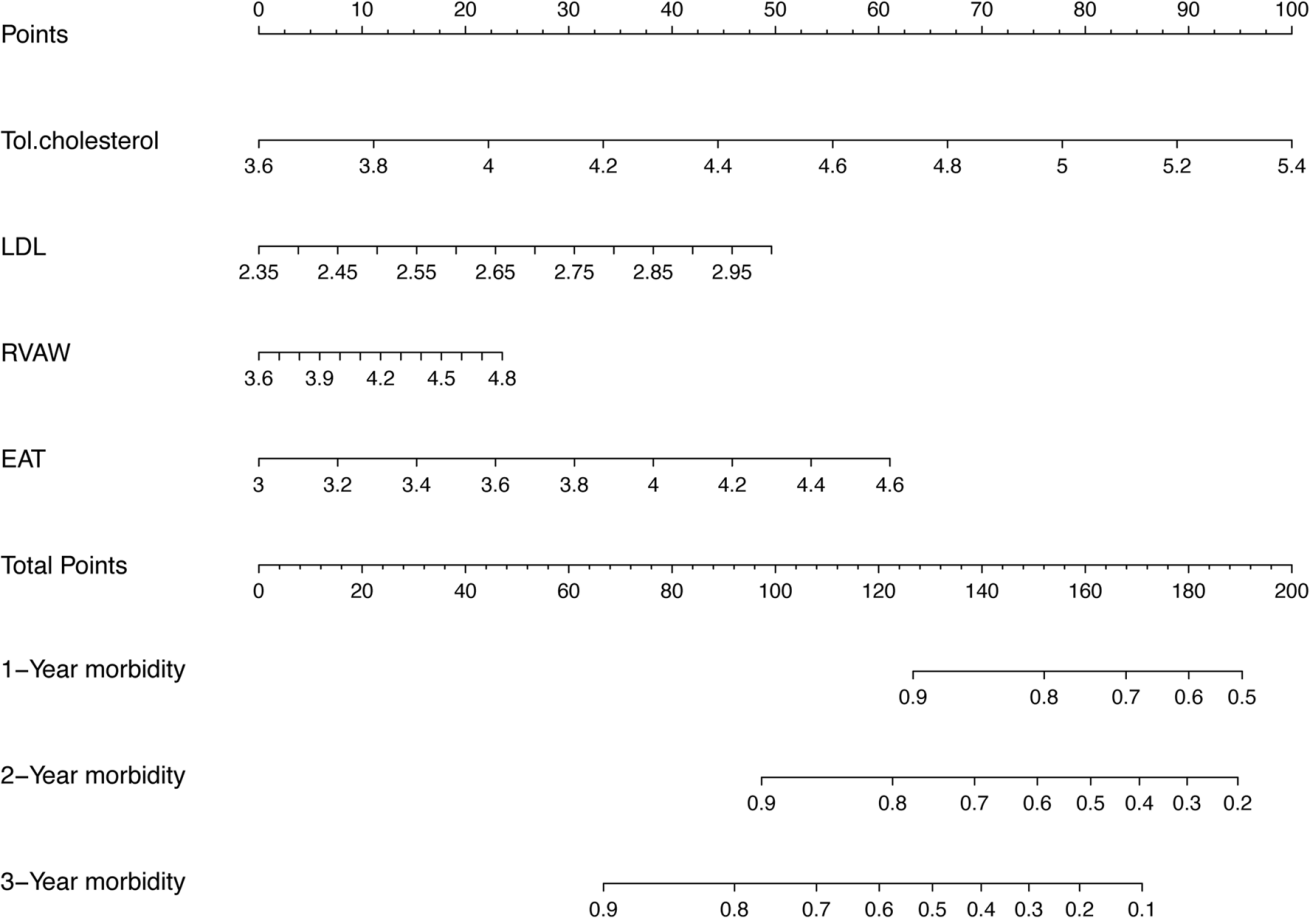
Nomogram constructed for predicting LVDD in patients with T2DM. *T.chol* total cholesterol, *LDL* low-density lipoprotein, *RVAW* right ventricular anterior wall, *EAT* epicardial adipose tissue

The LASSO method, a shrinkage estimation method, was used to select proper features in high dimensionality dataset. Through constructing a penalty function, we obtained compression coefficients and set them to zero to build a regression model. This study calculated risk scores from linear factors weighted by the coefficients and constructed a coefficient profile plot (Fig. 3A). Figure 3B showed a plot of cross-validated error in the LASSO linear regression model with a cross-validated error within 1 standard error of the minimum, and the model included 4 of the 19 variables. As shown in Fig. 4, the ROC curve analysis demonstrated that the AUC value for the EAT prognostic signature was 0.799, SBP (AUC = 0.793), Tol.chol (AUC = 0.800), LDL (AUC = 0.860), and RVAW (AUC = 0.827). These data demonstrate that the EAT prognostic signature is an independent prognostic factor for LVDD in T2DM subjects. A model incorporating 4 independent predictors (EAT, SBP, Tol.chol, LDL, and RVAW) was developed into a flow rate nomogram (Fig. 5).

## Discussion

In 2009, the first set of guidelines for echocardiographic evaluation of LVDD was published by ASE (American Society of Echocardiography) and EACVI (European Association of Cardiovascular Imaging)13. More revently in 2016, diastolic dysfunction ASE guidelines was updated factoring in several echocardiographic characteristics to diagnose and stage LVDD in an elegant fashion [14]. In previous trials, it has been showed that EAT thickness is associated with LVDD in metabolic syndrome patients [5]. To our knowledge, this is the first research to construct a simple-to-use nomogram which predicts the risk of LVDD in subjects with T2DM. The developed nomogram using data from the LASSO) logistic regression incorporates 4 items, EAT, SBP, Tol.chol, LDL, and RVAW. This novel nomogram could be applied into the outpatient setting to conduct a rapid assessment of LVDD risk in patients with T2DM. The extensive use of this nomogram in T2DM patients could potentially avoid excessive medical inspection, thereby lightening the burden on family's financial.

In this study, it was found that EAT mass was greater in subjects with T2DM, especial in LVDD group, which was consistent with previous studies [19–21]. The result indicated that EAT thickness is correlated with the E/A index in subjects with E/A ≥ 1. EAT is component of the visceral adipose tissue with both systemic and local effects around the heart. Leonetti confirmed that EAT thickness on echocardiography was found higher in subjects with more than one clinical and metabolic characteristics of Metabolic syndrome than those healthy [19]. A positive linear correlation was reported between EAT thickness and several parameters such as glucose and LDL cholesterol, especially fasting insulin [19, 22]. Our experimental results also found that some parameters have statistical association with EAT, such as HDL and fasting insulin.

In 2003, Iacobellis et al. [15] firstly proposed the echocardiography for EAT measurement. Echocardiography boasted unique advantages over computed tomography (CT) or magnetic resonance imaging (MRI). It is cheap and easily obtained, accompanied by high repeatability and fast application in clinical work. The research reported significant correlation with EAT and several clinical features, such as waist circumference, intracardiac fat and intra-abdominal fat [19, 23]. The amelioration of statistical modeling have induced the progress of nomograms to assess obstructive sleep apnea (OSA) risk depended on clinical features, demographic characteristics and somatometric measurement. Xu et al. [24] reported a nomogram which contained 8 independent factors (sex, age, glucose, insulin, apolipoprotein B, BMI, waist circumference and neck circumference) constructed by a LASSO regression method, that and predicted non-, moderate-to-severe and severe OSA accuracy. However, no questionnaires have been constructed for the purpose of identifying patients at high risk of LVDD prioritize potential treatment.

Since large amounts of subjects with T2DM encountered by endocrinologists, opening a window of opportunity to screen T2DM subjects on endocrinology wards for associated comorbidities, including LVDD, and when necessary, make appropriate interventions. In this study, the nomogram performed as a screening tool to identify T2DM patients at risk of LVDD, as demonstrated by AUC values > 0.6. To reduce overfitting, the 4-factor nomogram was built based on a LASSO model.

Epidemiological statistics showed chronic illness is the main reason of illness and death of middle-aged and aged people [25, 26]. In terms of morbidity and morbidity, T2DM and cardiovascular diseases are the top five chronic diseases among people worldwide [27]. In current research, we assessed the potential relationship between clinical parameters and cardiac function. The bivariate analysis result display close connection between HDL and EAT in subjects with LVDD. Fasting insulin has consistently shown significant association with EAT in group with normal LVDF. After considering associated medical comorbidities, Cavalcante et al. discovered EAT volume as an independent predictor of impaired LVDF [4]. Eun Park et al. [5] reported that EAT volume is independent correlate of LVDF in patients with metabolic syndrome. In a longitudinal study of Olmsted County, LVDD was demonstrated to increase all-cause mortality after adjusting for clinical characteristics [28]. The article focuses on diabetic patients, which is a supplement to published research results.

## Limitation

Echocardiographic is a linearity measurement in the same plane and can not measure EAT volume accurately. Study did not consider the patient's medication and course of diabetes. Adipokines such as tumor necrosis factor-α, interleukin-6, resistin should be measured, which can be better to analysis of the relationship between EAT and LVDF. EAT was assessed by echocardiography, which can only indirectly reflect the volume. The accuracy rate is lower than gold standards of CT and MRI. Due to the small sample size, we did not establish a training cohort and a validation cohort to verify the model.

## Supplementary Information

Below is the link to the electronic supplementary material.

Supplementary file1 (XLSX 35 KB)

## Abbreviations

BMI: Body mass index
SBP: Systolic blood pressure
DBP: Diastolic blood pressure
EAT: Epicardial adipose tissue
HbA1C: Glycated hemoglobin
HDL: High-density lipoprotein
LDL: Low-density lipoprotein
T. chol: Total cholesterol
RVAW: Right ventricular anterior wall
RVLW: Right ventricular lateral wall
LVPW: Left ventricular posterior wall
LVDF: Left ventricular diastolic function
LVDD: Left ventricular diastolic dysfunction
